# The Study of Chemical Profile and Antioxidant Properties of Poplar-Type Polish Propolis Considering Local Flora Diversity in Relation to Antibacterial and Anticancer Activities in Human Breast Cancer Cells

**DOI:** 10.3390/molecules27030725

**Published:** 2022-01-22

**Authors:** Michał Miłek, Ewa Ciszkowicz, Monika Tomczyk, Ewelina Sidor, Grzegorz Zaguła, Katarzyna Lecka-Szlachta, Anna Pasternakiewicz, Małgorzata Dżugan

**Affiliations:** 1 Department of Chemistry and Food Toxicology, Institute of Food Technology and Nutrition, University of Rzeszów, Ćwiklinskiej 1a, 35-601 Rzeszow, Poland; mwesolowska@ur.edu.pl (M.T.); ewelina.sidor.dokt@gmail.com (E.S.); apast@ur.edu.pl (A.P.); 2 Department of Biotechnology and Bioinformatics, Faculty of Chemistry, Rzeszow University of Technology, Powstańców Warszawy 6, 35-959 Rzeszow, Poland; eciszkow@prz.edu.pl (E.C.); szlachta@prz.edu.pl (K.L.-S.); 3 Doctoral School, University of Rzeszow, Poland, Rejtana 16c, 35-959 Rzeszow, Poland; 4 Department of Bioenergy, Food Analysis and Microbiology, Institute of Food Technology and Nutrition, University of Rzeszow, Zelwerowicza 4, 35-601 Rzeszow, Poland; g_zagula@univ.rzeszow.pl

**Keywords:** propolis, anticancer activity, antioxidants, polyphenols, HPTLC, antibacterial effect

## Abstract

Nine samples of ethanolic extracts of poplar-type propolis (EEP) originated from South-Eastern Poland were analyzed in terms of the diversity of the flora around the apiary. The mineral composition, antioxidant properties, polyphenolic profile (HPTLC), and main polyphenolic constituents (HPLC-DAD) were determined. Only minor differences in chemical composition and antioxidant capacity between tested EEPs were found regardless of their botanical origin. However, the biological activity of the EEPs was more diversified. The tested EEPs showed stronger antibacterial activity against Gram-negative bacteria (*Escherichia coli*) compared to Gram-positive bacteria (*Staphylococcus aureus* and *Staphylococcus epidermidis*). Staphylococci biofilm inhibition occurred as a result of exposure to the action of four out of nine EEPs (P1–P4). Due to the various compositions of individual EEPs, a different MCF-7 cellular response was observed according to inhibition of cells migration and proliferation. Almost every sample inhibited the migration of breast cancer cells at a low concentration (0.04 µg/mL) of propolis. Even at the lowest concentration (0.02 µg/mL), each EEP inhibited the proliferation of MCF-7 cells, however, the level of inhibition varied between samples.

## 1. Introduction

Propolis (bee glue) is a natural product collected by honeybees (*Apis mellifera* L.) from different plant organs, mainly leaves, buds, and exudates. The collected material is partially digested with the use of enzymes from the saliva of bees and mixed with beeswax to obtain a resinous consistency [1]. The role of propolis in a beehive is to protect the community from predators, seal the crevices and holes in honeycombs, and stabilize the temperature in the hive. The color of propolis varies from dark-brown, yellow, green, to red, depending mainly on its geographical origin and plant sources [1]. According to the plant origin and major constituents, seven types of propolis can be distinguished: Poplar, Birch, Green (alecrim), Red, Clusia, Pacific, and Mediterranean [2]. Although more frequently two propolis groups were differentiated: one from temperate regions and another from tropical areas, with completely different chemical characteristics [3,4]. The most popular type of propolis in Europe, North America, and the non-tropical regions of Asia is the poplar type. It is made from various species of poplars (*Populus* sp.), most commonly *P. nigra*. The chemical composition of propolis depends on the species of bees, geographic and climatic factors, and the collecting season. An important factor is the composition of the local flora that determines the classification of propolis of the appropriate type [1].

The latest reports on the chemical profile of propolis list hundreds of identified compounds in its composition [5,6]. The polar fraction, which includes aromatic acids, esters, and flavonoids, originates from bee metabolism or from contamination with honey (sugars). The non-polar components of propolis, mainly fatty acids and their esters, come from beeswax [7]. Propolis is rich in macro- and microelements, i.e., calcium, magnesium, potassium, sodium, iron, zinc, manganese, aluminum, barium, strontium, chromium, and chlorine. The bioelement content in propolis is about 0.6%. Vitamins B1, B2, B6 as well as vitamin C and vitamin E were also identified in propolis [8].

The most important fractions among phenolic compounds are acids, derivatives of hydroxybenzoic acid (gallic, gentisic, protocatechuic, salicylic, vanillic), and derivatives of hydroxycinnamic acid (p-coumaric, caffeic, and ferulic). Caffeic acid phenethyl ester (CAPE) is considered a major constituent of temperate zone propolis. This metabolite exhibits a broad spectrum of bioactivity, including inhibition of nuclear factor κB, inhibition of cell proliferation, induction of cell cycle arrest, and apoptosis. Flavonoids, such as pinobanksin, pinocembrin, galangin, chrysin, kaempferol, and quercetin, are common constituents of poplar-type propolis [9]. The preliminary bioactivity screening of natural compounds in propolis could be achieved with using high-performance thin-layer chromatography (HPTLC), and high-performance liquid chromatography (HPLC) [10,11,12,13], which are simple and rapid techniques for the identification of bioactive compounds present in extracts.

For a long time, the chemical composition of propolis was considered constant, and it was definitely not an object study of differences depending on the botanical composition or geographic origin. In most of the studies on the chemical composition and biological activity of propolis, the propolis samples tested were not characterized in terms of their origin. Although the composition of propolis strongly depends on geographical origin, its overall percent composition remains stable: resins and balsams (45–55%), waxes (8–35%), essential oils and aromatic (5–10%), fatty acids (5%), pollen (5%) and organic and mineral substances (5%). Due to insolubility in water, the most commonly used form of propolis is the ethanolic extract of propolis, however, other solvents were also used resulting in different compositions of extracts [14].

Despite the differences in the chemical compositions of propolis extracts, all of them exhibit broad spectrum biological activity, including antiseptic, antifungal, astringent, antioxidant, diastolic, anti-inflammatory, anesthetic immunomodulatory, as well as antiallergenic properties [15,16]. Propolis shows a broad spectrum of action against various bacteria, although its effect on microorganisms is not the same. Bactericidal and bacteriostatic effects are caused by the synergistic action of many components of propolis, which are still identified. Gram-positive bacteria are much more sensitive to propolis extracts than Gram-negative bacteria. However, all mentioned properties have made propolis an attractive ingredient in functional foods that are useful in ameliorating the symptoms and manifestations of metabolic syndrome and its associated chronic diseases [17,18].

The aim of the study was to characterize for the first-time poplar-type propolis samples originated from South-Eastern Poland in terms of their mineral composition and polyphenolic profile, as well as biological activity measured by antioxidant, antibacterial and anticancer action. Furthermore, the impact of the local flora surrounding the apiary on propolis activity was also discussed.

## 2. Results and Discussion

### 2.1. Mineral Composition

The concentrations of selected elements determined in the tested propolis are presented in Table 1. Among the microelements analyzed, the lowest average concentration was recorded for Mo—0.02 mg/100 g, while the highest was for Zn—1.37 mg/100 g. The concentration of the microelements analyzed was comparable with the results of Woźniak et al. [19] who tested Polish propolis, and with Dogan et al. [20] and Tosic et al. [21] who tested foreign samples. Among the macronutrients analyzed, the highest concentration of potassium (62.60 mg/100 g) and decreasingly P and Ca, both on average 36 mg/100 g was determined. Subsequently, S, Fe and Mg, and the lowest concentration for Na was found, on average 2.18 mg/100 g with high variability (93%). The tested macroelements, especially Na and Mg, were significantly lower compared to the other authors’ findings [19,20,21,22] whereas similar K and Ca was reported by Dogan et al. [20] and Fe by Woźniak et al. [19]. The average Cd and Pb content in the tested propolis samples was detected at 0.01 and 0.10 mg/100 g, respectively. Compared to Tosic et al. [21] and Abdullah et al. [22] such concentrations are very low. Nickel was tested by other authors at lower levels while Al was determined by them at comparable concentrations [21,22].

The studies of the authors who compared the mineral content in propolis samples from different regions, even within Poland [19,23], indicate that the concentrations of elements are very variable, and these differences are probably caused by the building material of the hive, the method of collecting the sample and the surroundings of the apiary [24]. This causes large differences in the mineral content between bee products from different locations. For example, differences in the concentration of zinc in propolis from different regions of the world can be as high as 400%. Similarly, large differences, reaching 130%, were found in the iron content of propolis from different apiaries [23]. Moreover, propolis is transformed much less by bees than wax and honey, and therefore reflects environmental contamination with more accuracy. On the other hand, it can be expected that high contamination of propolis with toxic metals will translate into increased contamination of honey and wax. Fortunately, propolis is used in very small amounts as human dietary supplements and is therefore not a serious source of toxic metals [24].

### 2.2. Antioxidant Capacity

For dry extracts of propolis samples, the total content of phenolic compounds and flavonoids, as well as antioxidant activity, was determined using the FRAP, DPPH, and ABTS methods (Table 2). The results obtained indicate a high content of phenolic compounds in all propolis extracts. Phenolic compounds are known to constitute one of the most important groups of bioactive substances in propolis, especially the fraction of phenolic acid derivatives and flavonoids [6,25]. The share of the flavonoid fraction in the total phenolic content is significant, up to 56% in the case of sample 9.

It is commonly known that the phenolic content of propolis should be distinguished from this for propolis extracts. Raw propolis, which may contain impurities, contains an average of 148 mg GAE/g of raw propolis [26] up to 359.1 mg GAE/g [27]. The content of metabolites depends on the method of obtaining propolis from the hive and the extraction system used for analysis [27,28]. When considering the dry propolis extract, the data obtained by us are in line with other authors’ findings. According to various reports, the ethanolic extract of Polish propolis (EEP) contains from 137.19 mg GAE/g of extract [29], to 220.05–275.79 mg GAE/g of extract [30]. For flavonoids, the contents of 18.76 up to 93.13 mg QE/g for 70% ethanol extract of Polish propolis were reported [30,31]. For propolis from Mexico, up to 379 mg QE/g of EEP was recorded [32].

The tested propolis extracts show a high antioxidant capacity. It is well known that due to the high content of secondary metabolites, mainly polyphenols, propolis is highly active against reactive oxygen species [33,34,35]. Among the samples tested, those with the highest level of phenols showed the highest antioxidant activity were samples 1, 3, and 9. The variety of methods and ways of expressing antioxidant activity by different authors does not allow direct comparisons of the results; however, the strong antiradical potential tested with the DPPH and ABTS assays was confirmed [36,37,38], as well as the reducing ability expressed by the FRAP method [39].

### 2.3. HPTLC and HPLC-DAD Polyphenolic Profiles

**Figure 1:**
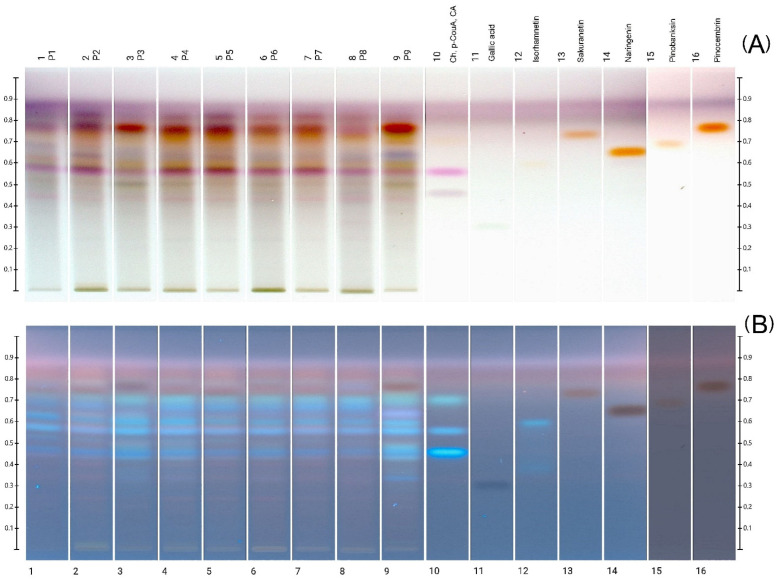
Images of HPTLC polyphenolic profile development after p-anisaldehyde derivatization, in visible light (**A**) and 366 nm UV light (**B**). Track 10 for the mixture of 3-components, in order of increasing Rf: CA—caffeic acid, p-CouA—p-coumaric acid, Ch—chrysin. P1–P9—propolis samples.

The polyphenol profiles of all the propolis extract samples obtained by the HPTLC method were abundant in various compounds (Figure 1). As a result of derivatization with p-anisaldehyde, an image was obtained consisting of colored bands, mainly yellow, orange, pink, blue in visible light (Figure 1A) and blue and green-blue in UV light (Figure 1B).

The dominant compounds include caffeic acid (Rf = 0.46), p-coumaric acid (Rf = 0.56), pinocembrin (Rf = 0.77) and isorhamnetin (Rf = 0.60), the bands of which are present in all samples. The deep blue-green band (in UV) at Rf = 0.72 appears to be chrysin, however, its presence in all samples was not confirmed by HPLC-DAD. It is possible that bands from other compounds overlap. Among the polyphenolic standards used, it was not possible to detect only gallic acid (Rf = 0.33) in the samples. In terms of qualitative composition, all profiles were similar; slightly more bands were observed for samples 3 and 9. In both samples, there is an intense band at Rf = 0.65, stained with a bluish-violet color (in visible light Figure 1A) and glowing bright purple in UV (Figure 1B). In sample 9 there are also visible additional bands surrounding the caffeic acid band, the intensity of which in the remaining samples is much weaker. The richer polyphenol profile of the above-mentioned samples correlates with a higher content of total polyphenols and flavonoids.

The HPTLC technique was previously used to characterize propolis samples by other authors. Polyphenol profiles of samples of different origins were compared to determine the type of propolis (O, B, and G-type) originating from Germany [11]. This technique was also used to determine the authenticity of European propolis samples, based on the characteristic marker compounds of the flavonoid group, e.g., naringenin, chrysin, galangin [40]. HPTLC polyphenol profiles were also useful for assessing the quality of commercial products based on propolis [13]. This technique allows direct comparison of the quality profiles of many samples at the same time; however, its limitation is the detection of the main, dominant components of the analyzed samples. Obtaining a complete profile, as in HPLC methods, especially those coupled with MS, is not possible without the use of appropriate standards. However, HPTLC allows the obtain of fingerprints that can be used to confirm identity easily, whereas the complete profile analysis, including quantitative analysis, is carried out using LC/MS techniques [13].

The polyphenol profiles of the propolis extract samples obtained by the HPLC-DAD method (Table S1) mainly include phenolic acids and flavonoids compounds. The dominant metabolites, present in all samples, were caffeic, p-coumaric, ferulic, benzoic acids, as well as compounds belonging to flavonoids: naringenin, pinobanksin, pinocembrin, and their derivatives (Table S1). Among the components identified by comparison with analytical standards, differences between samples were detected. Cinnamic acid was found in extracts number 3, 4, and 9 and in trace amounts in samples 2 and 5, only. 4-hydroxybenzoic acid was present in small amounts only in extracts 6–8. Chrysin was found only in samples 1 and 5, sakuranetin in samples 2, 4, 6, 7, 8 and apigenin only in sample 9. The mentioned compounds are commonly found in propolis of various origins; their presence or absence may be associated with the botanical origin of the sample. According to the literature data [41,42], the most common type of propolis in Poland (temperate zone of Europe) is poplar-type, originating from *Populus nigra* and *P. tremula*, as well as *Betula pubescens*. In comparison between the two poplar species, chrysin was present only in *P. nigra*, as were the pinocembrin and pinobanksin derivatives. Aspens (*P. tremula*) extracts contain other compounds such as glycerides and sakuranetin [15,42]. Among the plants that grow in the vicinity of the apiaries from which samples P1–P5 were obtained, there are many abundant in polyphenols. In addition to the typical sources of propolis, such as poplars and birches, there are other trees, such as willows (*Salix* sp.), lindens (*Tilia* sp.), fruit trees, and conifers. These compounds can come from many plant sources, i.e., flowers, leaves buds and plant sap, and it is generally accepted that the main role in the formation of propolis is played by leaf buds [1]. The buds of many trees contain numerous polyphenols, including various flavonoids and phenolic acids. Certain compounds are species-specific and their presence in propolis could indicate the use of these plants by bees, e.g., quercetin in larch buds, hyperoside and chlorogenic acid in ash buds, and catechin in pine buds [43]. Phenolic acids (caffeic, ferulic, benzoic, p-coumaric) dominated in the analyzed EEPs may most likely come from poplar buds but also from other deciduous trees in which they are present, e.g., *Betula* sp., *Prunus* sp., *Quercus* sp. [44,45,46] and also conifers (*Pinus* sp., *Picea* sp., *Abies* sp.) [47]. Similarly, in the case of flavonoids (chrysin, pinobanksin, pinocembrin, naringenin, sakuranetin) identified in EEPs, their occurrence has been reported in many plant buds [43,48].

For selected compounds, quantitative analysis by HPLC-DAD was performed. The results are summarized in Table 3. Quantitative HPLC-DAD analysis confirms that p-coumaric acid is the dominant metabolite in all tested EEP samples. Its content ranged from 3.46 µg/g for sample 8 to 46.75 for sample 2. The similar contents of this compound in Polish propolis extracts are provided by Grecka et al. [15]. In the samples where chrysin was detected (3 and 9), it was the highest among the quantified compounds. The high content of chrysin and sakuranetin in EEP is also confirmed in the literature [15]. Among the HPLC quantified polyphenols, ferulic and benzoic acids, as well as chrysin and pinocembrin, shaped the antioxidant activity of EEPs, regardless of the method used (Pearson’s correlation coefficients in the range of 0.5–0.8).

**Figure 2:**
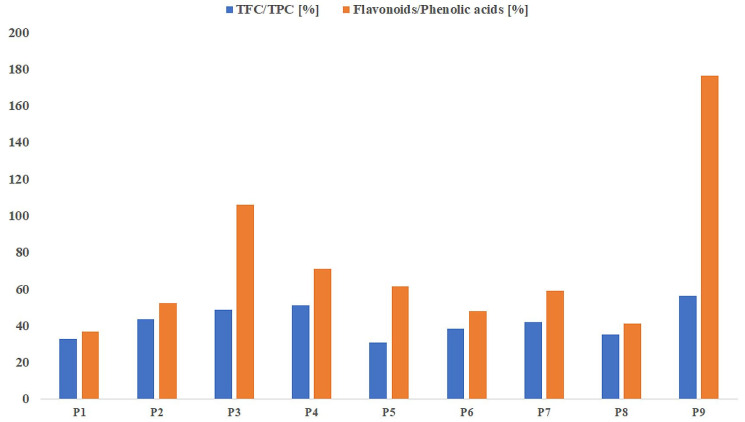
Percentage of the flavonoid fraction in the total content of phenolic compounds and the ratio of quantified flavonoids to phenolic acids (HPLC method). P1–P9—propolis samples.

The share of flavonoids in the total content of phenolic compounds and the ratio of the sum of flavonoids to the sum of phenolic acids determined by the HPLC-DAD method can be considered as an indicator of the activity of the propolis extract (Figure 2). Among the samples tested, only in the case of extracts 3 and 9, the share of the flavonoid fraction in the total content of polyphenols was at least 50%. Moreover, for both samples, the ratio of flavonoids to phenolic acids quantified by HPLC was greater than 1 (105.7 and 176.4%, respectively). These two samples were characterized by the strongest antioxidant properties; hence, we can risk the statement that it is the flavonoid fraction that determines the biological activity of propolis. The content of flavonoids is one of the postulated parameters for the standardization of propolis preparations [49]. A positive correlation was observed between the proposed indicators (r = 0.794). The high content of HPLC quantified flavonoids, especially pinobanksin, pinocembrin, and chrysin in samples P3 and P4, may indicate the main share of poplar excretions, which are a confirmed source of these compounds [15,42].

### 2.4. Antibacterial Activity Assays

To investigate the efficiency of propolis, the antibacterial properties of nine South-eastern Polish EEPs were assessed. The results are summarized in Table 4. The results show differential MIC values, which is a measure of the degree of antimicrobial activity. The lower the MIC concentration, the greater the antimicrobial activity. The most resistant bacteria turned out to be Gram-positive MRSA 4236 and Gram-negative *E. coli*, obtaining MIC values 390.6–781 µL/mL and 195.3–781 µg/mL, respectively.

As a result of the MBC test, the nature of the inhibition of bacterial growth was described as bacteriostatic, as none of the nine EEP samples, even at the highest concentration, was able to kill any of the bacterial strains.

Only certified samples P1–P4 were able to inhibit biofilm formation by *S. aureus* 6538 and *S. epidermidis* 35984, while P2–P4 showed the highest activity against *S. epidermidis* 35984 (24.4–48.8 µg/mL). Any EEP samples were able to inhibit biofilm formation of relevant clinical resistant bacterial strain (MRSA or MRCNS). Furthermore, there was no positive correlation between the content of individual polyphenols (Table 3) and the antibacterial activity expressed by the MIC value (data not shown).

According to literature reports [15,50], Gram-positive bacteria are more susceptible to propolis than Gram-negative, which is confirmed by the lower MIC values obtained for staphylococci than for *E. coli*. For ethanolic extracts of Polish propolis, effective activity against bacteria of the genus *Staphylococcus* has been demonstrated with MIC values between 32 and 256 μg/mL [15]. The activity of propolis against various strains of staphylococci was also confirmed in combination with other antimicrobial drugs, silver nanoparticles, and with honey [51,52,53]. The antimicrobial activity of propolis has also been shown to depend strongly on climatic factors, which determine the botanical origin and type of propolis [1,54], as well as the extraction conditions used [55]. In view of the growing resistance to known antibiotics, recognition of the factors determining the antibacterial effect of propolis may be of key importance for the use of this natural substance in the treatment of bacterial infections, which has been intensively studied in many laboratories around the world [56,57]. Recently, the properties of EEPs against viruses, including the SARS-CoV-2 coronavirus, have also been extensively studied [58,59].

### 2.5. Cancer Cells Migration Inhibition Assay

**Figure 3:**
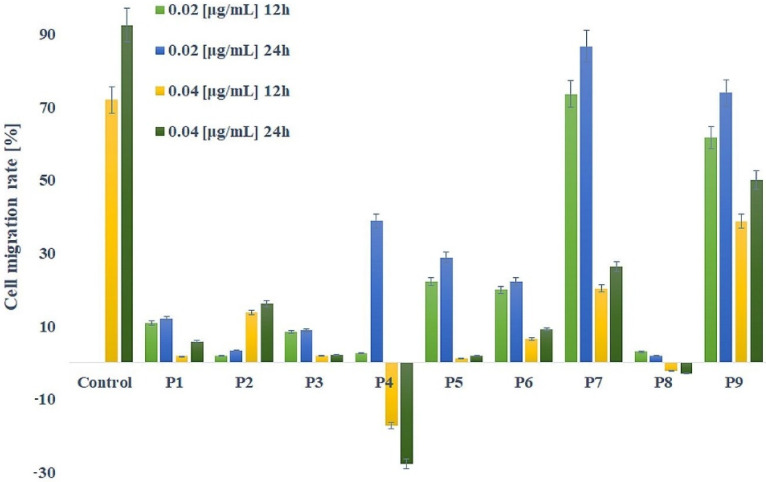
Inhibition of MCF-7 cells migration after incubation with P1–P9 propolis samples determined with the scratch test. Data are presented as means ± SD. P1–P9—propolis samples.

To evaluate the influence of the propolis samples tested on the migration of MCF-7 cells, a scratch test was performed at 12 and 24 h. The validity of the scratch wound healing assay was already confirmed as a method of choice to test the ability of cancer cells to migrate toward the applied wound [60,61]. By using microscopic images, it was possible to evaluate the evolution of the gap created in the confluent cell monolayer in the presence of the samples. Treatment with propolis, at concentrations 0.02 and 0.04 µg/mL, significantly decreased cell migration in comparison with the control at 12 h and 24 h (Figure 3, Table S2).

The effectiveness to inhibit the migration of MCF-7 cells induced by a lower dose of propolis (0.02 µg/mL) after 12 h of incubation decreased in order: P2 > P4 > P8 > P3 > P1 > P6 > P5 > P9 > P7. In these concentration samples P7 (after 12 and 24 h) and P9 (only after 12 h) did not show a significant difference (*p* > 0.05) compared to the untreated control, as they do not influence the migration of MCF-7 cells through the wound.

The highest activity in inhibiting cell migration established P4 and P8 (in both concentrations and after 12 and 24 h), P3 and P5 (0.04 µg/mL after 24 h of treatment). Interestingly, the P4 and P8 samples caused the scratch to expand at both concentrations, which may indicate such a strong effect of these samples that they additionally caused a cytotoxic effect.

Only sample P2 showed a direct proportional dependence of concentration and time on the percentage of wound closure: 1.78% and 3.21% by incubation with 0.02 µg/mL, 13.74% and 16.07% by incubation with 0.04 µg/mL, respectively after 12 and 24 h (Figure 3). Treatment of all other samples led to a higher wound closure migration rate of at lower concentrations, therefore 0.04 µg/mL inhibits the migration of cancer cells to a greater extent, which is an advantageous result in terms of metastasis formation. The diversified results obtained may be due to more than 300 constituents of propolis samples (including benzoic acid, cinnamic acid, flavonoids) [62], which probably causes interactions between compounds of a synergistic, additive, or antagonistic nature. Inhibition of cancer cell migration by propolis extracts and individual components has been confirmed, among others, for glioblastoma, prostate, colorectal, lung, bladder, as well as breast cancer cells (MCF-7) [63,64,65,66,67,68].

After prolonged incubation (24 h), the inhibition of cancer cells migration activity of all samples, except P4 and P8, decreased with time, thus the highest inhibition of cell migration is observed after 12 h. The P9 sample showed the highest percentage of scratch closure, reaching 73.99% of the migration rate of MCF-7 cells after 24 h of incubation. Increased migration of MCF-7 cells (compared to other EEP samples) confirms the results of Darbre et al. (2013), where exposure of MCF-7 cells to aluminum ions induced migration, motility, and wound healing of breast cancer cell lines [69], since P9 is the sample with the highest aluminum ion concentration obtained by the ICP-OES method. However, the molecular mechanisms, responsible for the ability of aluminum ions to increase migratory and invasive properties of MCF-7 cells have not yet been identified.

**Figure 4:**
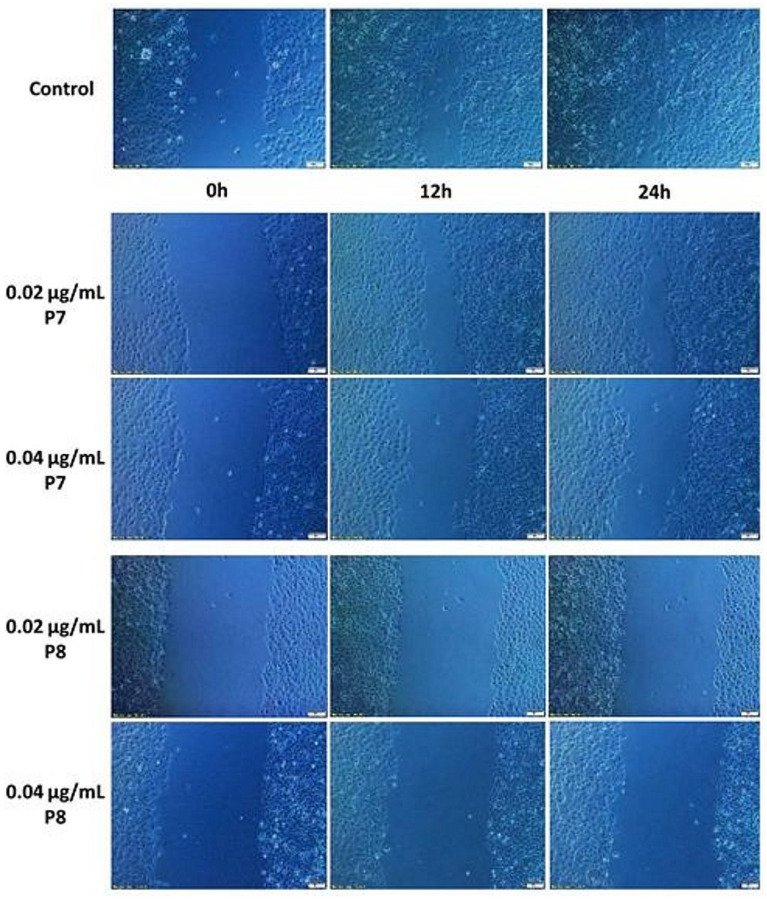
Selected microscopic images of the MCF-7 cell migration scratch assay as a result of 12 and 24 h of exposure to EEPs of P7 and P8 samples.

Selected microscopic photos showing the migration process of MCF-7 cells not treated with propolis extract (control) and with slightly inhibited migration (P7) and strongly inhibited migration, with cytotoxicity (P8) are shown in Figure 4.

### 2.6. Cell Proliferation Assay

**Figure 5:**
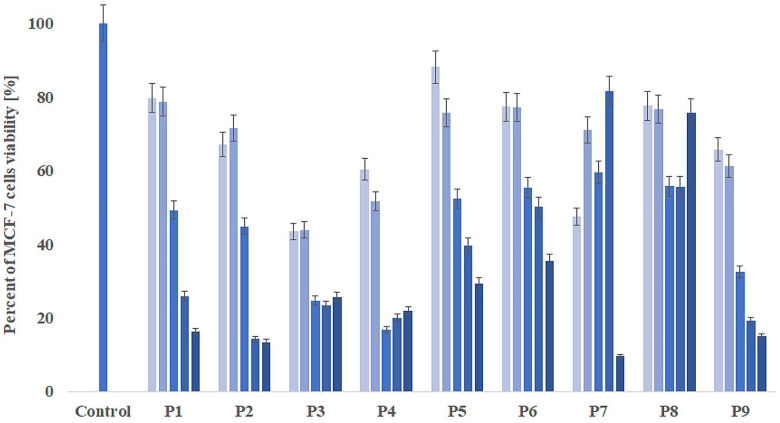
Inhibitory effects against MCF-7 cells at each concentration of 9 different EEP samples after 24 h of incubation. The color reflects the concentration of the sample (from lightest to darkest): 0.04, 0.08, 0.2, 0.4, 0.8 µg/mL. Data are presented as means ± SD. P1–P9—propolis samples.

To determine the cytotoxicity of 9 propolis samples, MCF-7 was treated with P1–P9 at different concentrations (0.04, 0.08, 0.2, 0.4, 0.6 µg/mL) (Figure 5). Then, the WST-1 assay was performed to define the concentration that causes a 50% decrease in cell viability (IC_50_, µg/mL). MCF cells treated with propolis samples showed a decline in cell viability and proliferation in a dose-dependent manner, for P1, P5, P6, and P9. The statistically significant IC_50_ values were calculated only for P1, P2, P5, P6, and P9, and were, respectively, 0.26, 0.19, 0.33, 0.39, and 0.14 µg/mL (IC_50_ for cisplatin was 5.1µg/mL). As a result of the presence of compounds such as phenolic acids and flavonoids, tested EEP samples could inhibit breast cancer cell proliferation. Surprisingly, the lowest IC_50_ value was observed for P9, which caused the highest cell migration rate, and thus the lowest activity in inhibiting the migration of MCF-7 cells. It can be due to different compounds in the P9 propolis sample, including a 3-times higher level (compared to other studies EEPs) of toxic Al ions (17.07 mg/100 g), which are responsible for migration and proliferation mechanisms in breast cancer cells. Some studies indicate that aluminum ions may induce DNA damage [70], subsequently increasing cytotoxicity in MCF-7, without influence on normal human lung fibroblast or primary rat hepatocytes [71]. The beneficial flavonoids apigenin [72] and chrysin [73,74] with documented anticancer properties, were only identified, respectively, in samples P9 and P3/P9, respectively. However, in P9, the concentration of chrysin was 1.5 times higher compared to P3, which may also explain the high cytotoxicity (IC_50_ = 0.14 µg/mL) of this sample. No concentration-dependent effect was observed on inhibition of MCF-7 cell proliferation for P3, P4, P7, and P8. This may be due to the complexity of the composition of propolis and possible interactions between individual components, which make it impossible to obtain results dependent on the concentration of total propolis samples. Further analysis of the cellular and molecular mechanisms underlying the anticancer indications of the studied EEP samples must be performed.

It is already known that propolis is extremely cytotoxic to cancer cells and non-toxic to normal human skin fibroblasts [75,76], thus our results are in line with those findings. For MCF-7 cells, it was shown that standardized extracts of Turkish poplar-type propolis, containing mainly 3-O-methylquercetin, chrysin, caffeic acid, CAPE, galangin, and pinocembrin, induced cell cycle arrest and resulted in accumulation in the G0/G1 phase of cancer cells [77]. The effectiveness of EEPs against various cancer cells, for example, leukemia, osteosarcoma, glioblastoma, breast adenocarcinoma, melanoma, prostate cancer, has been demonstrated in vitro [64,78,79], indicating the possibility of using this preparation in the treatment of neoplastic diseases.

## 3. Materials and Methods

### 3.1. Chemicals

Chemicals (2,2-diphenyl-1-picrylhydrazyl; 2,2′-azino-bis(3-ethylbenzothiazoline-6-sulfonic acid); 2,4,6-Tris(2-pyridyl)-s-triazine), reagents (Folin–Ciocalteu reagent), standards: caffeic acid, ferulic acid, benzoic acid, 4-hydroxybenzoic acid, p-coumaric acid, apigenin, sakuranetin, chrysin, naringenin, taxifolin, pinobanksin, pinocembrin were obtained from Sigma Aldrich (Saint Louis, MO, USA), and buffer components (chloroform, ethyl acetate, formic acid, ethanol, acetonitrile, formic acid) were purchased from Avantor Performance Materials Poland SA (APM, Gliwice, Poland).

### 3.2. Media and Bacterial Strains

Mueller Hinton Broth (MHB, NutriSelect^®^ Plus, pH 7.4, Merck Millipore, Burlington, MA, USA), Mueller Hinton Agar (MHA, NutriSelect^®^ Plus, pH 7.3, Merck Millipore, Burlington, MA, USA), phosphate buffered saline (pH 7.4), oxacillin sodium salt monohydrate (OXA, >97%) were purchased from Sigma Aldrich St. Louis, MO, USA. Tetracyclin-hydrochlorid (TET, ≥95%), gentamycin sulphate (GEN), 3-(4,5-dimethyl-2-thiazolyl)-2,5 diphenyl-2H-tetrazolium bromide (MTT, ≥98%) and ethanol (EtOH, ≥98%) were purchased from Carl Roth GmbH+ Co., Karlsruhe, Germany. Gram-negative: Escherichia coli ATCC 10536 (*E. coli*) and Gram-positive: *Staphylococcus aureus* ATCC 6538 (*S. aureus*) and *Staphylococcus epidermidis* ATCC 12228 (*S. epidermidis* 12228; does not form a biofilm) were used as a part of the collection of the Department of Biotechnology and Bioinformatics, Faculty of Chemistry, Rzeszow University of Technology, Rzeszow, Poland. Gram-positive Staphylococcus epidermidis ATCC 35984 (*S. epidermidis* 35984; forming a biofilm) was obtained from the Chair and Department of Medical Microbiology Medical University of Lublin, Lublin, Poland. Clinical Methicillin-Resistant *Staphylococcus aureus* (MRSA 4236) and clinical Methicyllin-Resistant Coagulase-Negative Staphylococci *Staphylococcus epidermidis* (MRCNS 2452) were obtained from the Department of Medical Laboratory Diagnostics of Provincial Specialist Hospital in Rzeszow, Rzeszow, Poland.

### 3.3. Material Collection

Nine samples of propolis were studied, five of them (no. 1–5) came from apiaries with a known location, the rest (no. 6–9) were purchased on the local market, specifying of their exact origin. For samples no. 1–5, information was collected on the dominant vegetation in the immediate vicinity of the apiaries based on the beekeeper’s declaration. These data are summarized in Table 5.

### 3.4. Preparation of Propolis Dry Extract

One gram of each propolis sample was poured with 10 mL of 70% ethanol. The samples were shaken in the dark for 24 h at 400 rpm. After this time, propolis samples were placed in ultrasound for 20 min, 40 mHz, and then filtered through a filter paper. The extracts were subjected to the condensation process to remove ethanol (RVC 2–18 CDPlus, Martin Christ, Osterode am Harz, Germany), frozen to −65 °C, and then freeze-dried (Alpha 1–2 LD plus, Martin Christ, Osterode am Harz, Germany) to obtain dry extract. For the determination of antioxidant activity, chromatographic analyzes and antimicrobial effect study, extracts with a concentration of 100 mg/mL were prepared.

### 3.5. Mineral Composition of Bioelements Using the ICP-OES Method

The evaluation of selected minerals (Na, K, Ca, Mg, P, S, Fe, Mn, Zn, Cr, Cu, Sr, As) and the toxic metals (Al, Cd, Pb) was determined by optical emission spectrometry with inductively-induced plasma (ICP-OES) using a Thermo iCAP 6500 spectrophotometer (Thermo Fisher Scientific Inc., Waltham, MA, USA). The detection limit for each element was determined at a level that was not less than 1 µg/L. A curve fit factor for the elements studied was above 0.99. All the analyses were made in three independent replications for each sample. The targeted repeatability expressed as the relative standard deviation (RSD) and the targeted recovery ranged from 92% to 106%, respectively. The method was validated using certified reference material (INCT-TL-1 tea leaves and NIES CRM No. 7 Tea Leaves). The response of the equipment was periodically checked with known standards. To identify the relevant measurement lines and avoid possible interferences, the method of adding an internal standard was applied. Yttrium and ytterbium ions were used as internal standards.

### 3.6. Antioxidants Assay

DPPH Radical Scavenging Activity was measured based on the procedure described by Dżugan et al. [80]. Briefly, 0.02 mL of appropriate propolis extract was added to 0.18 mL of 0.1 mM DPPH (Sigma Aldrich Co., St. Louis, MO, USA) solution in methanol (Sigma Aldrich Co., Sain Louis, MO, USA), and left in dark for 30 min. Then, the absorbance was measured at 517 nm using a UV–VIS Spectrometer (EPOCH 2 microplate spectrophotometer, BioTek, Winooski, VT, USA). The results obtained were expressed as µmol Trolox (Sigma Aldrich Co., St. Louis, MO, USA)—Trolox equivalents (TE) equivalents per 1 g of the dry weight of the extract based on the prepared standard curve (25–300 nmol/mL of Trolox solution in methanol).

FRAP Assay (ferric reducing antioxidant power) was also provided according to Dżugan et al. [80]. Briefly, 0.02 mL of sample was mixed with 0.18 mL FRAP reagent, and the absorbance of the mixture was measured spectrophotometrically (EPOCH 2 microplate spectrophotometer) at 593 nm after 10 min of incubation at 37 °C against blank. A calibration curve was prepared for Trolox (Sigma Aldrich Co., St. Louis, MO, USA) ethanol solution in the range 25–300 nmol/mL, and the results were expressed as μmol of Trolox equivalents (TE) per 1 g of the extract.

ABTS (2,2′-azino-bis(3-ethylbenzothiazoline-6-sulfonic acid)) radical cations inhibition was measured according to the method of Re et al. [81] with slight modifications. Briefly, 0.02 mL of appropriate propolis extract was mixed with 0.18 mL of 0.1 mM ABTS solution and kept in the dark for 6 min. In the control sample, the extract was replaced by proper solvent. After incubation, the absorbance of the test and control samples was measured at 734 nm in a microplate reader (EPOCH 2 microplate spectrophotometer). Results were expressed as μmol Trolox equivalents (TE) per 1 g of extract (μmol/g) from the calibration curve prepared for Trolox in the range 25–300 nmol/mL.

### 3.7. Total Phenolic (TPC) and Flavonoid (TFC) Content

The total phenolic content was also measured using the procedure described by Dżugan et al. [80]. In summary, 0.02 mL of plant extract was mixed with 0.1 mL Folin–Ciocalteu reagent (diluted 10×) and next 0.08 mL of 7.5% (*w*/*v*) sodium carbonate solution was added. After incubation at room temperature for 60 min, the absorbance was measured spectrophotometrically (EPOCH 2 microplate spectrophotometer) at 760 nm against the blank. TPC was calculated based on a calibration curve at the range 25–150 µg/mL. Results were expressed as mg of gallic acid equivalents (GAE) per 1 g of the extract.

The total flavonoid content (TFC) was assessed using the method of Biju [82]. In summary, 0.1 mL µL of the extract was mixed with 0.1 mL 2% AlCl_3_ (in methanol). The reaction mixture was incubated for 10 min at room temperature until the reaction was complete. The absorbance of the solution was then measured at 415 nm with a microplate reader EPOCH 2 against methanol blank. The total content of flavonoids in the extracts of was expressed in mg of quercetin equivalent (QE) per g of dry extract (mg QE/g d.e.). The results were calculated based on a calibration curve prepared for quercetin in the range 0–125 µg/mL.

### 3.8. Polyphenolic Profile by the HPTLC Method

Analysis of all obtained ethanolic extracts from propolis samples was performed on HPTLC Silica Gel 60 F_254_ plates (20 cm × 10 cm) purchased from Merck (Darmstadt, Germany). Extracts (2 µL) were applied to the plate as 9 mm bands from the lower edge of the plate at a rate of 100 nL/s using a semi-automated HPTLC application device (Linomat 5, CAMAG, Muttenz, Switzerland).

The chromatographic separation was carried out in a chromatographic tank saturated for 20 min with the mobile phase and developed to a distance 70 mm. The results obtained were documented using an HPTLC imaging device (TLC Visualizer, CAMAG) under white light, UV 254, and 366 nm. In addition, each plate was derivatized using an automated derivatizer of TLC plates (CAMAG Derivatizer) with p-anisaldehyde reagent. After derivatization, the plates were imaged under white light and 366 nm. The obtained chromatographic images were analyzed using HPTLC software (Vision CATS, CAMAG, Muttenz, Switzerland).

### 3.9. Identification of Polyphenols by HPLC-DAD Method

Analyses were performed on a Gilson chromatographic system (Gilson Analytical-to-Semipreparative HPLC System, Gilson Inc., Middleton, WI, USA) equipped with a binary gradient pump (Gilson 322), a column thermostat (Knauer, Berlin, Germany), autosampler with a fraction collector (Liquid Handler GX-271, Gilson Inc., Middleton, WI, USA) and a photodiode array detector (DAD, Gilson 172, Gilson Inc., Middleton, WI, USA). The analytical column (Poroshell 120, EC C-18, 4.6 × 150 mm, Agilent Technologies Inc., Santa Clara, CA, USA), thermostated at 40 °C, was used for the chromatographic separation. The mobile phase (1 mL/min) consisted of 0.1% (*v*/*v*) formic acid in water (phase A) and acetonitrile (phase B). The samples (10-fold diluted) were eluted by the following gradient: 10% B (1.5 min), 10–100% B (1.5–20 min), 100% B (20–25 min), and again 10% B to equilibrate column. The injection volume was 10 μL. The chromatograms were recorded at 254, 280, 320, and 360 nm. Phenolic compounds were identified and classified into the specific groups by their UV–VIS spectra, literature data, and by comparison of their retention time values with values of standards. External standards were used for quantitative analysis, including caffeic acid, ferulic acid, p-coumaric acid, benzoic acid, chrysin, sakuranetin, pinocembrin, pinkobanksin, and naringenin. Data were expressed using calibration curves at concentrations ranging from 25 to 400 µg/mL (R^2^ ≥ 0.96). The results were expressed as mg/g of the dry weight of the extract (mg/g DW).

### 3.10. Antibacterial Activity Assays

All bacterial strains were grown from frozen stocks and subcultured at least twice before use in experiments to ensure normal growth patterns at 37 °C in New Brunswick Innova 40 Shaker (Eppendorf AG, Hamburg, Germany). All reagents and bacterial cultures were prepared using Laminar Flow Cabinet ESCO Airstream (Esco Lifesciences GmbH, Friedberg, Germany). The antibacterial activity of nine EEPs against the four bacterial strains was evaluated by determining of the minimum inhibitory concentrations (MIC, µg/mL) using the micro-broth dilution method in MHB, as described before [83]. After the MICs were determined, the minimum bactericidal concentrations were assessed by MHA agar plating method. In order to determine the anti-biofilm activity of tested EEPs, the MTT method was performed. The initial bacterial culture was the same for all antibacterial activity assays used. Briefly, after 24 h of incubation at 37 °C in New Brunswick Innova 40 Shaker (Eppendorf AG, Hamburg, Germany), the number of cells in suspension was adjusted to the 0.5 McFarland standard (10^8^ colony-forming units, CFU/mL) using BIO-RAD SmartSpec^TM^ Plus Spectrophotometer (Hercules, CA, USA), λ = 630 nm.

A series of two-fold EEPs dilutions was prepared on 96-well plate in MHB, obtaining a concentration in the range from 0.76 µg/mL to 6.25 mg/mL. An appropriate bacterial culture at 105 CFU/mL density was added to the prepared series of solution dilutions and incubated at 37 °C for 24 h. Then bacterial growth was monitored and the lowest concentration of the antibacterial agent was defined, which completely inhibited the visible growth of the microorganism (MIC). A positive (the medium without antibacterial agents) and negative control (no bacterial cultures added) of bacterial growth and solvent control (70% EtOH) were performed. The determination of MBC was performed based on the MIC results by plating 100 µL of the mixture of bacteria in the environment of MIC, 2xMIC, 4xMIC, and 8xMIC concentrations. After 24 h of incubation at 37 °C, the plates were assessed by manual counting of the colonies formed that corresponded to a single bacterial cell in the mixture.

After assessing the MIC and MBC assays, the anti-biofilm test was performed. Medium from 96-well plates was removed and washed twice with sterile (PBS) to remove the planktonic bacteria. Alive and adherent bacterial cells that usually formed biofilm in each well of the microtiter plate were stained with MTT (MTT; 0.5% in PBS) for 2 h at 37 °C (protected from light) [84]. After incubation, the solution was removed, and the bacterial biofilm was solubilized by DMSO and mixed for 15 min at room temperature in an INNOVA 40 Incubator Shaker. The concentration at which no solubilized formazan was observed was indicated as biofilm inhibitory concentration (µg/mL).

### 3.11. Cancer Cell Culture and Propolis Treatment

Human MCF-7 breast cancer cells were kindly provided by Professor Zbigniew Madeja, Department of Cell Biology, Jagiellonian University, Cracov, Poland. The MCF-7 cell line was cultured in 75 cm^2^ flasks (Nunc^TM^ EasYFlask^TM^, ThermoFisher Scientific, Roskilde, Denmark) in complete Eagle’s Minimum Essential Medium (EMEM) with 10% fetal bovine serum (FBS). The dissociation of MCF-7 adherent cells was performed with Trypsin-EDTA solution and the cells were counted using the TC20 Automated Cell Counter by trypan blue staining. The propolis samples were prepared in EMEM, so that the final concentration of the solvent (70% ethanol) was 0.3%. The final concentrations of all propolis samples were 0.02 µg/mL and 0.04 µg/mL. All reagents and culture media were obtained from American Type Culture Collection (ATCC, Manassas, VA, USA).

### 3.12. Cancer Cells Migration Inhibition Assay

The scratch wound-healing assay was adapted in order to analyze the inhibition of MCF-7 cancer cells migration by modifying the protocol of Governa et al. [85]. Briefly, MCF-7 cells were seeded into twelve-well cell culture plates (5 × 10^4^ cells/well) and allowed to grow as a monolayer in 37 °C and 5% CO_2_. After reaching 90% of confluence, a 200-µL pipette tip was used to scratch two straight lines in the middle of the well. Cells were washed with Dulbecco’s Phosphate Buffered Saline (D-PBS, ATCC, VA, USA) and a fresh medium with 5% FBS and treatments was added to each well. Immediately after scratching and after 12 and 24 h, images were obtained in the same regions with the use of an Olympus IX83 inverted microscope [86]. The experiments were conducted until the untreated scratched cells, served as control, reached approximately 100% of confluence. All experiments were carried out in triplicate and in two independent series, obtaining six repetitions (*n* = 6). The images were analyzed by ImageJ, an open-source image processing program, and the percentage of MCF-7 cells migration into the wound was calculated as shown below in Equation (1):

(1) $$Cell\;Migration\;\left[\%\right]=\left(\frac{A_{t=0}-A_{t=\Delta t}}{A_{t=0}}\right)\times 100\%$$

where *A_t_*_=0_ is the initial scratch area and *A_t_*_=Δ*t*_ is the scratch area after *n* hours of the initial scratch, both in µm^2^.

### 3.13. Cell Proliferation Assay

Cell proliferation evaluation was performed using the WST-1 assay (Abcam, Cambridge, UK) in accordance with the manufacturer’s instructions. Briefly, MCF-7 cells were seeded into 96-well plates (1 × 10^4^ cells/well) and incubated in 37 °C and 5% CO_2_ for 24 h. Fresh medium was added with an appropriate amount of each propolis sample, thus obtaining final concentrations of 0.04, 0.08, 0.2, 0.4 and 0.6 µg/mL. The positive control was a known anticancer agent, cisplatin in the range of concentration between 1 and 100 µM. After 24 h of incubation, 10 µL of WST-1 reagent was added to each well, the plates were incubated for 2 h in culture conditions and placed on a microliter plate shaker for 1 min at room temperature. The spectrophotometric measurement of absorbance was performed with a microplate reader (BioRad, Hercules, CA, USA) at a wavelength of 490 nm versus 630 nm to eliminate background factors. The blank samples were wells without cells, treated with WST-1 according to the above-mentioned protocol. Treatments were performed in triplicate in two independent experiments.

### 3.14. Statistical Analysis

All calculations were made in triplicate, unless otherwise indicated. For the data obtained, mean values have been calculated as well as the standard deviation. Significant differences (*p* < 0.05) between samples were calculated using Tukey’s test. The correlation between some parameters was calculated using Spearman’s correlation rank. All calculations were made using Statistica 13.3 software (StatSoft, Tulsa, OK, USA).

## 4. Conclusions

In conclusions, poplar-type propolis originated from South-eastern Poland is an abundant source of polyphenols which resulted in its high antioxidant activity. Despite the differences found in chemical composition and activity, the impact of flora surrounding the apiary was not confirmed. It has been demonstrated that Polish poplar-type propolis exhibit antibacterial properties, especially against Gram-positive bacterial strains. Moreover, selected samples were able to inhibit the biofilm formation of certified *S. aureus* and *S. epidermidis*, which is relevant in the treatment of increasing antibiotic resistance and preventing chronic infections. The beneficial effect of EEPs on the migration and proliferation of breast cancer cells was determined using scratch test cell migration assay for the first time. However, the strict relationship between the chemical compositions of propolis and its biological activity was not fully recognized. However, the greater the share of flavonoids in the total polyphenols content in ethanolic extracts was observed, the better the antibacterial activity was found. The recognition of key factors that influence the biological activity of propolis extracts is the crucial issue for their application in health prophylaxis and food preservation. Due to promising observations obtained, further studies are required, especially in the field of EEPs standardization and new formulations development.

## Figures and Tables

**Table 1:** Mineral composition of the studied propolis samples (P1–P9) obtained by ICP-OES with prior microwave mineralization.

	P1	P2	P3	P4	P5	P6	P7	P8	P9	Min	Max	Mean	SD	Variability%	F-Value	*p*-Value
microelements [mg/100 g]																
Mo	0.01	0.02	0.02	0.01	0.02	0.03 *	0.01	0.01	0.02	0.01	0.03	0.02	0.01	42.54	14.4	0.000
Sr	0.04 *	0.07 *	0.09 *	0.10	0.07 *	0.11	0.16 *	0.18 *	0.18 *	0.04	0.18	0.11	0.05	45.45	2315.6	0.000
Cu	0.13 *	0.13 *	0.18	0.20	0.17	0.25	0.21	0.20	0.45 *	0.13	0.45	0.21	0.10	44.69	117.4	0.000
Cr	0.78	0.81	0.68	0.64	0.75	0.69	0.73	0.74	0.79	0.64	0.81	0.73	0.06	7.53	8.3	0.000
Mn	0.37 *	1.05	0.71 *	0.96	0.87	0.65 *	1.12	1.82 *	1.20 *	0.37	1.82	0.97	0.41	41.96	269.0	0.000
Zn	0.09 *	1.04 *	0.80 *	0.99 *	0.38 *	0.73 *	0.76 *	5.42 *	2.14 *	0.09	5.42	1.37	1.62	118.15	148,834.5	0.000
macroelements [mg/100 g]																
Na	0.34 *	1.19 *	0.62 *	0.97 *	5.62 *	0.77 *	1.49 *	5.06 *	3.53 *	0.34	5.62	2.18	2.02	92.88	1122.5	0.000
Mg	4.29 *	9.65 *	12.82	12.09 *	12.95	12.98	13.23	19.08 *	19.65 *	4.29	19.65	12.97	4.60	35.46	3896.2	0.000
Fe	12.05 *	11.55 *	10.75 *	11.32 *	13.97	16.84	13.62	17.20	30.03 *	10.75	30.03	15.26	6.01	39.36	392.5	0.000
S	4.85 *	10.65 *	13.92 *	13.83 *	15.45 *	15.23 *	22.73 *	22.86 *	28.27 *	4.85	28.27	16.42	7.11	43.29	4402.8	0.000
Ca	7.24 *	17.31 *	28.53 *	37.01	33.57	46.27 *	40.51	56.17 *	59.22 *	7.24	59.22	36.20	16.99	46.94	1248.9	0.000
P	8.89 *	27.06 *	27.86 *	24.24 *	48.26	13.30 *	45.00	61.87 *	71.19 *	8.89	71.19	36.41	21.43	58.85	281.8	0.000
K	15.99 *	54.21 *	67.89 *	60.19	61.79	89.13 *	62.86	68.66 *	82.64 *	15.99	89.13	62.60	20.68	33.03	2764.9	0.000
toxic elements [mg/100 g]																
Cd	n.d.	0.01	0.01	0.01	0.01	n.d.	0.01	0.01	0.01	0.00	0.01	0.01	0.00	36.77	15.5	0.000
Pb	0.03 *	0.04 *	0.03 *	0.03 *	0.03 *	0.05 *	0.03 *	0.52 *	0.17 *	0.03	0.52	0.10	0.16	157.58	1482.7	0.000
Ni	0.54	0.51	0.53	0.51	0.58	0.51	0.52	0.50	0.70 *	0.50	0.70	0.54	0.06	11.47	503.3	0.000
Al	4.54 *	4.95 *	5.82 *	5.30 *	6.95	7.85	7.51	8.22	17.07 *	4.54	17.07	7.58	3.80	50.12	1847.5	0.000
Total	37.23	104.08	130.66	127.29	151.19	157.12	156.45	195.95	231.00							

* Statistically significant differences (*p* < 0.05) between the sample and the mean. n.d.—below detection level (<1 ppm).

**Table 2:** Total phenolic and flavonoids content and antioxidant properties of dried extracts (d.e.) of tested propolis samples.

Sample Number	TPC [mg GAE/g d.e.]	TFC [mg QE/g d.e.]	FRAP [μmol TE/g d.e.]	DPPH [μmol TE/g d.e.]	ABTS [mmol TE/g d.e.]
P1	289.29 *	94.42 *	681.25 *	351.40 *	7.77 *
P2	263.84 *	114.05 *	485.53 *	219.48 *	5.72 *
P3	319.20 *	155.11 *	699.34 *	357.63 *	6.31 *
P4	285.12 *	144.96 *	581.25 *	252.56 *	6.35 *
P5	278.72 *	85.04 *	566.12 *	231.55 *	6.00
P6	252.38 *	96.87 *	489.14 *	212.09 *	4.75 *
P7	267.86 *	112.14 *	493.09 *	230.38 *	6.02
P8	268.90 *	93.82 *	533.22 *	224.15 *	5.25 *
P9	326.34 *	183.62 *	725.99 *	342.84 *	6.02
Min	252.38	85.04	485.53	212.09	4.75
Max	326.34	183.82	725.99	357.63	7.77
Mean	283.52	120.03	583.88	269.12	6.02
SD	24.95	33.77	95.38	62.22	0.83
Variability [%]	8.80	28.13	16.34	23.12	13.83
F-value	6.52	73.36	14.61	28.13	11.77
*p*-value	0.005	0.000	0.000	0.000	0.001

d.e.—dry extract. * Statistically significant differences (*p* < 0.05) between the sample and the mean.

**Table 3:** Quantitative data of selected polyphenol content in propolis samples determined by HPLC-DAD.

Sample Number	Caffeic Acid [µg/g of Extract]	*p*-Coumaric Acid [µg/g of Extract]	Ferulic Acid [µg/g of Extract]	Benzoic Acid [µg/g of Extract]	Sum of Phenolic Acids [µg/g of Extract]	Chrysin [µg/g of Extract]	Naringenin [µg/g of Extract]	Sakuranetin [µg/g of Extract]	Pinobanksin [µg/g of Extract]	Pinocembrin [µg/g of Extract]	Sum of Flavonoids [µg/g of Extract]
P1	0.44 *	38.36 *	15.95 *	22.65 *	77.4	nd *	0.84 *	20.46 *	2.13 *	5.04 *	28.47
P2	1.83 *	46.75 *	10.73 *	12.10 *	71.41	nd *	1.10 *	21.97 *	7.03 *	7.12 *	37.22
P3	2.16 *	22.78 *	13.08 *	19.33 *	57.35	39.61 *	0.15 *	nd *	7.29 *	13.57 *	60.62
P4	2.88 *	42.54 *	12.90 *	16.42 *	74.74	nd *	2.04 *	31.02 *	9.79 *	10.17 *	53.02
P5	1.43 *	37.88 *	10.62 *	18.65 *	68.58	nd *	1.54 *	28.17 *	5.00 *	7.92 *	42.06
P6	1.57	39.88 *	10.81 *	15.37 *	67.63	nd *	0.63 *	17.63 *	6.57 *	7.45 *	32.28
P7	1.28 *	38.31 *	9.83 *	9.36 *	58.78	nd *	0.75 *	18.98 *	7.84 *	7.17 *	34.74
P8	0.07 *	30.27 *	8.31 *	17.29 *	55.94	nd *	0.16 *	16.04 *	1.11 *	5.58 *	22.89
P9	2.52 *	13.88 *	12.26 *	17.36 *	46.02	61.49 *	nd	nd *	8.54 *	11.14 *	81.17
Min	0.07	13.88	8.31	9.36		0.00	0.00	0.00	1.11	5.04	
Max	2.88	46.75	15.95	22.65		61.49	2.04	31.02	9.79	13.57	
Mean	1.58	34.52	11.61	16.50		11.23	0.80	17.14	6.14	8.35	
SD	0.91	10.40	2.22	3.93		22.95	0.68	10.85	2.89	2.76	
Variability%	57.93	30.12	19.15	23.80		204.32	84.66	63.27	47.08	33.03	
F-value	33,323.11	4,324,609.00	197,680.00	617,202.00		21,071,411.00	18,401.44	4,705,307.44	334,762.11	304,370.44	
*p*-value	0.000	0.000	0.000	0.000		0.000	0.000	0.000	0.000	0.000	

nd—not detected. * Statistically significant differences (*p* < 0.05) between the sample and mean.

**Table 4:** Minimum inhibitory (MIC) and biofilm inhibitory values of propolis extracts against four certified bacterial strains and two clinical *S. aureus* and *S.* *epidermidis* strains; no MBC values were identified in the tested range of concentrations from 0.76 µg/mL to 6.25 mg/mL.

EEP Sample	*E. coli*	*S. aureus*		*S. epidermidis*		
		6538	MRSA 4236 ^a^	12228	35984	MRCNS 2452 ^a^
	MIC (μg/mL)					
P1	390.6	195.3/390.6 ^b^	781	195.3	195.3/781 ^b^	48.8
P2	195.3	24.4/390.6 ^b^	781	24.4	48.8/48.8 ^b^	24.4
P3	195.3	24.4/390.6 ^b^	781	48.8	48.8/48.8 ^b^	390.6
P4	390.6	24.4/390.6 ^b^	390.6	48.8	48.8/24.4 ^b^	24.4
P5	781	390.6/-	390.6	48.8	390.6/-	390.6
P6	390.6	390.6/-	390.6	48.8	195/-	48.8
P7	781	390.6/-	390.6	48.8	390.6/-	6.1
P8	390.6	390.6/-	390.6	195	390.6/-	24.4
P9	390.6	195.3/-	390.6	48.8	195.3/-	24.4
Tetracycline (positive control)	0.5	0.12/0.12	7.8	62.5	0.12/0.24	62.5

^a^—alert pathogen. ^b^—the concentration that completely inhibits the biofilm formation of a specific strain. ‘-’—no inhibitory action against bacterial biofilm.

**Table 5:** Botanical description of propolis samples based on the beekeeper’s declaration.

Sample Number	Localization of Apiary	Most Abundant Plants near the Apiary
P1	rural area (49°50′ N, 21°69′ E)	*Malus domestica*, *Pyrus communis*, *Ribes nigrum*, *Aronia melanocarpa*, *Alnus* sp., *Betula* sp., *Salix* sp., *Padus avium*, *Robinia pseudoacacia*, *Tilia* sp., *Brassica napus*, *Solidago* sp., *Impatiens* sp.
P2	rural area (49°79′ N, 21°94′ E)	*Abies* sp., *Picea* sp., *Quercus* sp., *Salix* sp., *Frangula* sp., *Tilia* sp., *Populus tremula*, *Betula* sp., fruit trees, *Taraxacum officinale*, *Mentha* sp., *Centaurea* sp., *Rubus* sp., *Eupatorium cannabinum*, *Solidago* sp., *Thymus pulegoides*
P3	rural area (49°54′ N, 21°97′ E)	*Abies* sp., *Pinus* sp., *Larix* sp., *Acer* sp., *Tilia* sp., *Betula* sp., *Fagus* sp., *Salix* sp., *Rubus* sp., meadow flowers, forest shrubs
P4	urban area (49°68′ N, 21°77′ E)	*Picea pungens*, *Thuja* sp., *Chamaecyparis* sp., *Tilia* sp., *Acer* sp., *Taraxacum officinale*, *Solidago* sp.
P5	rural area (49°67′ N, 21°80′ E)	*Pinus* sp., *Tilia* sp., *Carpinus* sp., *Salix* sp., *Robinia pseudoacacia*, *Quercus* sp., *Abies* sp., *Picea* sp., *Prunus avium*, *Thuja* sp., *Trifolium repens*, *Solidago* sp., meadow flowers, garden plants
P6	rural area (49°70′ N, 21°77′ E)	not specified
P7	rural area (49°78′ N, 22°54′ E)	not specified
P8	rural area (51°19′ N, 22°61′ E)	not specified
P9	rural area (49°95′ N, 20°81′ E)	not specified

## Data Availability

The data presented in this study are available in the article and supplementary material.

## Supplementary Materials

The following supporting information can be downloaded online, Table S1: Qualitative HPLC profiles of propolis extract samples; Table S2: In vitro MCF-7 cells migration rate as a result of exposure to EEPs compared to untreated control (% of cell migration ± standard deviation).

## Sample Availability

Samples of propolis extracts are available from the authors for the limited time.
