# Supplementary material for: The Study of Chemical Profile and Antioxidant Properties of Poplar-Type Polish Propolis Considering Local Flora Diversity in Relation to Antibacterial and Anticancer Activities in Human Breast Cancer Cells
Source: Molecules. 2022 Jan 22;27(3):725. doi: 10.3390/molecules27030725 (PMC8840218; doi:10.3390/molecules27030725)
Supplement: Supplementary file 1 [file molecules-27-00725-s001.zip › molecules-1540605-supplementary.pdf]

Article

The Study of Chemical Profile and Antioxidant Properties of Poplar-Type Polish Propolis Considering Local Flora Diversity in Relation to Antibacterial and Anticancer Activities in Human Breast Cancer Cells

Michał Miłek [1,*], Ewa Ciszkowicz [2], Monika Tomczyk [1], Ewelina Sidor [1,3], Grzegorz Zaguła [4], Katarzyna Lecka-Szlachta [2], Anna Pasternakiewicz [1] and Małgorzata Dżugan [1,*]

[1] Department of Chemistry and Food Toxicology, Institute of Food Technology and Nutrition, University of Rzeszów, Ćwiklinskiej 1a, 35-601 Rzeszów, Poland; mwesolowska@ur.edu.pl (M.T.);
ewelina.sidor.dokt@gmail.com (E.S.); apast@ur.edu.pl (A.P.)

[2] Department of Biotechnology and Bioinformatics, Faculty of Chemistry, Rzeszow University of Technology, Powstańców Warszawy 6, 35-959 Rzeszów, Poland; eciszkow@prz.edu.pl (E.C.);
szlachta@prz.edu.pl (K.L.-S.)

[3] Doctoral School, University of Rzeszow, Poland

[4] Department of Bioenergy, Food Analysis and Microbiology, Institute of Food Technology and Nutrition, University of Rzeszow, Zelwerowicza 4, 35-601 Rzeszow, Poland; g_zagula@univ.rzeszow.pl

Table S1. Qualitative HPLC profiles of propolis extract samples

Peak number	Retention time [min]	Absorption maxima [nm]	Identification	P1	P2	P3	P4	P5	P6	P7	P8	P9
**1**	4,59	256	4-hydroxybenzoic acid*	-	-	-	-	-	+	+	+	-
**2**	5,44	216, 244sh, 295sh, 325	Caffeic acid*	+	+	+	+	+	+	+	+	+
**3**	6,55	282	4-hydroxybenzaldehyde	+	+	+	+	-	+	+	+	+
**4**	7,28	211, 226, 310	*p*-coumaric acid*	+	+	+	+	+	+	+	+	+
**5**	7,94	218, 235, 294sh, 323	Ferulic acid*	+	+	+	+	+	+	+	+	+
**6**	8,35	218, 235, 294sh, 323	Isoferulic acid	+	+	+	+	-	+	-	+	+
**7**	9,19	230	Benzoic acid*	+	+	+	+	+	+	+	+	+
**8**	9,95	218, 235, 294sh, 323	Caffeic acid derivative	-	-	+	-	-	-	-	-	+
**9**	10,06	232, 285, 321	Benzoic acid derivative?	+	+	-	+	+	+	-	+	+
**10**	10,40	233, 257, 370	Isorhamnetin*	+	-	+	+	+	+	+	-	+
**11**	10,89	288	Flavanone derivative	-	-	+	+	-	+	-	+	+
**12**	11,14	205, 220, 278	Cinnamic acid*	-	+	+	+	+	-	-	-	+
**13**	11,44	268, 332	Apigenin*	-	-	-	-	-	-	-	-	+
**14**	11,46	228, 289, 323sh	Naringenin*	+	+	+	+	+	+	+	+	-
**15**	11,67	229, 270, 315, 364sh	Unknown	+	+	+	+	+	+	+	+	+
**16**	11,88	292	Pinobanksin*	+	+	+	+	+	+	+	+	+
**17**	11,95	226, 293, 324sh	Unknown	-	-	-	-	+	-	-	-	-
**18**	12,39	229, 312	Unknown	+	-	-	+	+	+	+	+	+
**19**	12,75	226, 300sh, 312	*p*-coumaric acid derivative	+	+	+	+	+	+	+	+	+
**20**	13,00	216, 244sh, 295sh, 325	Caffeic acid derivative	-	+	+	+	+	-	+	+	-

**21**	13,05	231, 312	Unknown	-	-	-	-	-	-	-	-	+
**22**	13,70	211, 226, 310	*p*-coumaric acid derivative	+	+	+	+	+	+	+	+	+
**23**	13,83	211, 226, 310	*p*-coumaric acid derivative	+	+	+	+	+	+	-	+	+
**24**	13,95	224sh,296sh, 316	Caffeic acid derivative	+	+	+	+	+	+	+	+	+
**25**	14,12	269, 320sh	Chrysin*	-	-	+	-	-	-	-	-	+
**26**	14,19	294, 324sh	Flavanone derivative	+	-	-	-	+	-	-	-	-
**27**	14,22	291, 327sh	Sakuranetin*	-	+	-	+	-	+	+	+	-
**28**	14,35	291	Pinocembrin*	+	+	+	+	+	+	+	+	+
**29**	14,43	269, 290, 360	Galangin derivative?	+	+	+	+	+	+	+	+	+
**30**	14,55	292	Flavanone derivative	+	+	+	+	+	+	+	+	+
**30**	15,37	211, 226, 310	*p*-coumaric acid derivative	+	+	+	+	+	+	+	+	+
**31**	15,53	211, 226, 310	*p*-coumaric acid derivative	+	+	+	-	-	-	+	+	+
**32**	15,68	234sh, 295sh, 320	Caffeic acid derivative	+	+	+	+	+	+	+	+	-
**33**	15,73	234, 295sh, 319	Caffeic acid derivative	-	-	-	-	-	-	-	-	+
**34**	15,89	226, 297sh, 318	Caffeic acid derivative	+	+	+	+	+	+	+	+	-
**35**	15,93	234, 295sh, 319	Caffeic acid derivative	-	-	-	-	-	-	-	-	+
**36**	16,40	289	Flavanone derivative	+	+	+	+	+	-	+	+	+
**37**	16,62	234, 254sh, 294sh, 312	*p*-coumaric acid derivative	+	+	+	+	+	+	+	+	+
**38**	16,91	292	Flavanone derivative	-	-	+	-	-	-	-	-	+
**39**	17,11	289	Flavanone derivative	+	+	+	+	+	+	+	+	+
**40**	17,33	289	Flavanone derivative	+	+	+	+	+	+	+	+	+
**41**	17,64	231, 268, 359	Galangin derivative?	+	+	+	+	+	+	+	+	+
**42**	17,96	290	Flavanone derivative	-	-	+	-	-	-	+	-	+
**43**	18,25	235, 273, 295sh	Unknown	+	+	+	+	+	+	+	+	+
**44**	18,50	278	Unknown	+	+	-	+	-	+	+	+	+

* - identification based on comparison with analytical standard

Table S2. *In vitro* MCF-7 cells migration rate as a result of exposure to EEPs compared to untreated control (% of cell migration ± standard deviation).

**Samples [µg/mL]**		**Cell migration [%]**			
		Mean ± SD	*p*-value	Mean ± SD	*p*-value
		12h		24h	
**Control***		72.05 ± 2.63	-	92.54 ± 1.82	**-**
**P1**	0.02	10.85 ± 0.88	< 0.0001	11.97 ± 1.17	< 0.0001
	0.04	1.73 ± 0.83	< 0.0001	5.84 ± 1.24	< 0.001
**P2**	0.02	1.78 ± 0.03	< 0.0001	3.21 ± 0.37	< 0.001
	0.04	13.74 ± 0.68	< 0.005	16.07 ± 0.64	< 0.001
**P3**	0.02	8.44 ± 0.18	< 0.001	8.77 ± 0.13	< 0.001
	0.04	1.8 ± 0.48	< 0.001	2.16 ± 0.17	< 0.001
**P4**	0.02	2.56 ± 0.42	< 0.001	38.89 ± 2.15	< 0.005
	0.04	-17.22 ± 1.9	< 0.01	-27.66 ± 1.4	< 0.001
**P5**	0.02	22.29 ± 1.89	< 0.001	28.78 ± 0.47	< 0.001
	0.04	1.15 ± 0.8	< 0.001	1.95 ± 0.00	< 0.001
**P6**	0.02	19.95 ± 2.92	< 0.005	22.19 ± 2.63	< 0.005
	0.04	6.47 ± 0.42	< 0.001	9.12 ± 1.28	< 0.001
**P7**	0.02	73.65 ± 4.65	**0.691**[a]	86.7 ± 3.39	**0.104** [a]
	0.04	20.33 ± 2.81	< 0.005	26.35 ± 0.7	< 0.001
**P8**	0.02	2.94 ± 1.15	< 0.0001	1.92 ± 0.79	< 0.0001
	0.04	-2.33 ± 0.51	< 0.001	-2.93 ± 0.51	< 0.001
**P9**	0.02	61.7 ± 4.82	**0.117** [a]	73.99 ± 4.78	< 0.05
	0.04	38.79 ± 0.65	< 0.005	50.15 ± 0.56	< 0.005

* - treatment without propolis samples

[a] – no statistical significance between tested and control group
